# Posttranscriptional Gene Regulation of the GABA Receptor to Control Neuronal Inhibition

**DOI:** 10.3389/fnmol.2019.00152

**Published:** 2019-06-25

**Authors:** Rico Schieweck, Michael A. Kiebler

**Affiliations:** Department of Cell Biology and Anatomy, Medical Faculty, Biomedical Center (BMC), Ludwig-Maximilians-University of Munich, Munich, Germany

**Keywords:** posttranscriptional gene regulation, GABA receptors, inhibitory synapse, co-translational folding/assembly, RNA binding, RNA transport, local translation, RNA-binding proteins

## Abstract

Behavior and higher cognition rely on the transfer of information between neurons through specialized contact sites termed synapses. Plasticity of neuronal circuits, a prerequisite to respond to environmental changes, is intrinsically coupled with the nerve cell’s ability to form, structurally modulate or remove synapses. Consequently, the synaptic proteome undergoes dynamic alteration on demand in a spatiotemporally restricted manner. Therefore, proper protein localization at synapses is essential for synaptic function. This process is regulated by: (i) protein transport and recruitment; (ii) local protein synthesis; and (iii) synaptic protein degradation. These processes shape the transmission efficiency of excitatory synapses. Whether and how these processes influence synaptic inhibition is, however, widely unknown. Here, we summarize findings on fundamental regulatory processes that can be extrapolated to inhibitory synapses. In particular, we focus on known aspects of posttranscriptional regulation and protein dynamics of the GABA receptor (GABAR). Finally, we propose that local (co)-translational control mechanism might control transmission of inhibitory synapses.

## Introduction

The enormous capacity of the brain to store information and respond to different environmental conditions and challenges crucially rely on underlying mechanisms like synaptic plasticity. This depends on the ability to modulate the strength of transmission between two nerve cells as well as the growth and removal of synapses. Synapses consist of (at least) hundreds of proteins that need to be organized and correctly assembled to ensure proper synaptic function. Changes in synaptic transmission and structure are accompanied and conveyed by local alterations in protein levels. Understanding the regulation of synaptic protein composition is, therefore, crucial to gain insight into complex neurological processes such as learning and memory and, eventually, into neuropsychiatric diseases such as autism spectrum disorders, schizophrenia and bipolar disorders.

In order to remodel the synaptic proteome, neurons exploit different mechanisms that allow spatial and temporal control of protein levels. Protein synthesis was one of the first molecular mechanisms that were discovered to be indispensable for memory formation (Hershkowitz et al., 1975; Shashoua, 1976). Pioneer experiments showed that inhibiting translation blocked the ability of an animal to remember after training (Flexner et al., 1963). In line with this observation, several experiments have shown that strengthening and weakening of synaptic transmission, so called long-term potentiation (LTP) and depression (LTD), respectively, need active translation in a time-dependent manner (Krug et al., 1984; Linden, 1996). The spatial selectivity of synapses to undergo changes upon stimulation raised the question of how a cell knows, which synapse is destined for functional and structural remodeling. This inspired Frey and Morris (1997) to the idea of “synaptic tagging.” Repetitive activation of synapses, therefore, equips such a synapse with a labile molecular “tag.” Eventually, the synaptic tag allows the synapse to recruit newly synthesized proteins. The concept of “synaptic tagging” is a very elegant model to explain processes such as LTP and LTD at excitatory synapses (Frey and Morris, 1997). The precise identity of the tag(s) is still lacking. Furthermore, synaptic plasticity depends on additional processes such as mRNA localization, which is mainly independent of translation activity (Steward et al., 1998). mRNA transport and localization are important determinants of synaptic function (Jung et al., 2014). To date, it is generally believed that mRNAs are assembled into ribonucleoprotein particles (RNPs) consisting of mRNAs and RNA-binding proteins (RBPs). The protein and mRNA composition of these particles differ substantially (Kanai et al., 2004; Fritzsche et al., 2013) giving raise to the idea that different subtypes of particles or granules co-exist in a nerve cell. The function of these RNA granules is: (i) to transport mRNA—in a translationally dormant stage—along cytoskeletal elements such as microtubules to their destination at the synapse; and (ii) to regulate the translation of their target mRNAs. Activity-dependent disassembly of these RNA granules then allows the release of mRNAs and subsequent induction of translation. How neuronal stimulation, recruitment of mRNAs and unpacking of RNPs are synchronized is largely unknown. A pioneer study identified the kinase mechanistic target of rapamycin (mTOR) as a central hub to recruit RNAs. The authors suggest that mTOR might be the tag that controls mRNA recruitment at the synapse (Sosanya et al., 2015). mTOR is essential for proper neuronal function (Costa-Mattioli and Monteggia, 2013; Pernice et al., 2016). It needs to be experimentally verified though whether it might represent an universal synaptic tag or whether it might be specific for a subset of mRNAs.

Local protein expression control comprising mRNA transport, local protein synthesis and recruitment of newly synthesized protein remodel the synaptic proteome. Consequently, protein degradation is compulsive to complete synaptic remodeling. Synaptic protein degradation is induced in an activity-dependent manner (Bingol and Schuman, 2006). Moreover, it is tightly linked to translation to balance the protein need (Klein et al., 2015). In line with this finding, the translation repressor poly(A)-binding protein interacting protein 2A (PAIP2A) is degraded by calpain in neurons upon stimulation (Khoutorsky et al., 2013). Interestingly, calpain also degrades gephyrin (Gphn), a major scaffold protein at inhibitory synapses (Tyagarajan and Fritschy, 2014). This finding indicates that translational activation at excitatory synapses may modulate inhibitory synapses to alter transmission.

In this review article, we provide insight into posttranscriptional regulatory mechanisms that control synaptic protein expression. Since most of these studies investigated these processes at excitatory synapses, we aim to expand these fundamental aspects to inhibitory synapses. We speculate that local expression control also regulates inhibitory transmission to balance neuronal excitation.

## To Localize or Not to Localize—It’s a Matter of RBP Binding to the 3′-UTR

**Figure 1:**
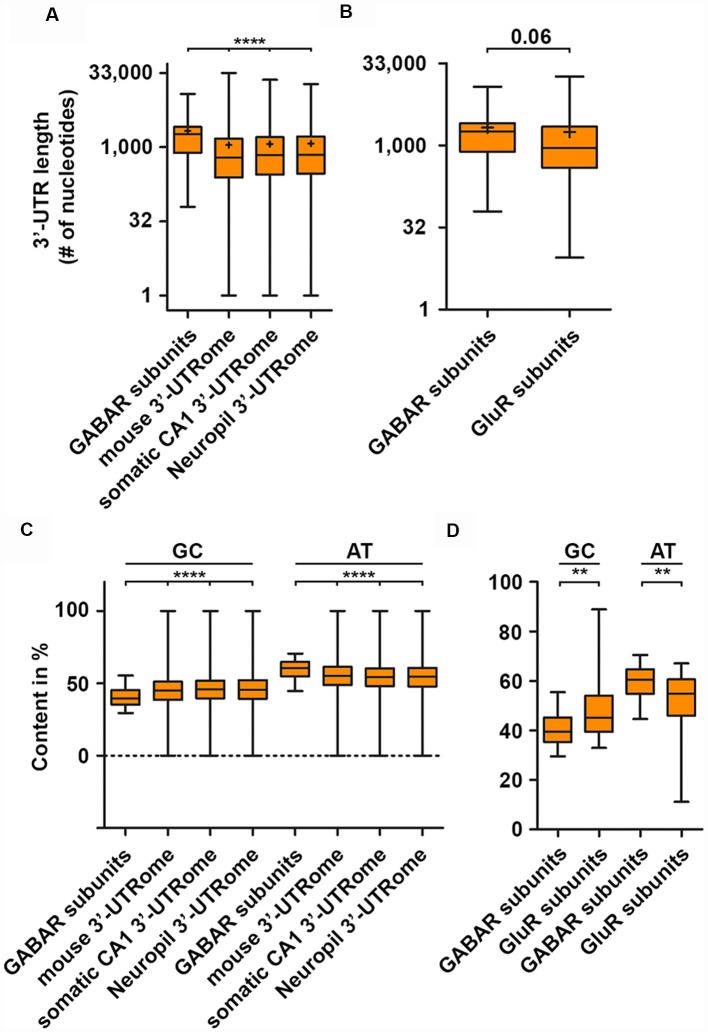
GABA receptor (GABAR) subunits exhibit extended 3′-untranslated region (3′-UTR) length. 3′-UTR lengths of GABAR (GABA_A_ and GABA_B_ receptor) subunits compared to the global mouse, hippocampal CA1, neuropil 3′-UTRome **(A)** and the 3′-UTR lengths of ionotropic GluR subunits **(B)**. GC and AT content of GABAR subunits 3′-UTRs compared to the global mouse, hippocampal CA1 and neuropil 3′-UTRome **(C)** as well as ionotropic GluR subunits **(D)**. Abbreviation: ^+^represents the mean. *P*-values were calculated using the Mann-Whitney *U*-test, **p < 0.01, ****p < 0.0001.

With the emergence of the individual-nucleotide resolution UV crosslinking and immunoprecipitation (iCLIP) technology (Huppertz et al., 2014), transcriptome-wide identification of RBP mRNA targets and binding site became experimentally addressable. iCLIP has now been performed for a series of RBPs (Tables s 1, 2). Interestingly, most of the RBP binding occurs within the 3′-untranslated region (3′-UTR) of transcripts (Andreassi and Riccio, 2009). In addition, it was shown that the median of the 3′-UTR length of mRNAs bound to the RBP Staufen2 that is necessary for RNA transport (Heraud-Farlow and Kiebler, 2014) is longer than the median of the transcriptome (Heraud-Farlow et al., 2013). This finding indicates that a certain 3′-UTR length is needed to allow association with RBPs and, consequently, mRNA transport and/or expression control (Heraud-Farlow and Kiebler, 2014). To test whether mouse GABA receptor (GABAR) subunits show a similar tendency towards longer 3′-UTR length, we analyzed the nucleotide length of their 3′-ends of all GABA_A_ and GABA_B_ receptor subunit isoforms (see “Methods” section). Strikingly, GABAR subunits reveal a significant increase in their 3′-UTR compared to the total mouse 3′-UTRome (Figure 1A). Moreover, the 3′-UTR length was significantly extended when comparing the GABAR subunits with the 3′-UTRome of the somatic and neuropil layer of the hippocampal CA1 region (Cajigas et al., 2012; Figure 1A). An increase in 3′-UTR length is linked with decreased translational activity in HEK cells and human neurons (Floor and Doudna, 2016; Blair et al., 2017) probably due to a higher number of miRNA and RBP binding sites. In addition, 3′-UTR length is extended during neuronal development indicating increased translation regulation in mature neurons compared to developing nerve cells (Blair et al., 2017). Of note, GABAR subunits exhibited a trend towards longer 3′-ends when compared with ionotropic glutamate receptor subunits (Figure 1B). Together, these results suggest that GABAR subunit 3′-UTRs have a high(er) potential to be bound by RBPs. Supportive for this hypothesis is the fact that GABAR subunit mRNAs are enriched in the dendrite containing neuropil layer of CA1 neurons in the hippocampus (Cajigas et al., 2012) suggesting that these mRNAs are localized there. The recognition of mRNA targets by RBPs relies on binding sites within their 3′-UTRs and that each mRNA might have its own specific RNA signature. In detail, these binding sequences consist of both sequence and structural elements (Kiebler and Bassell, 2006; Doyle and Kiebler, 2011; Jung et al., 2014; Sugimoto et al., 2015). Interestingly, GABAR subunits exhibited a lower GC content compared to the total, somatic CA1 and neuropil 3′-UTRome (Figure 1C). Concomitantly, we observed a higher AT content (Figure 1C). Moreover, the same statistically significant effects were detected when comparing ionotropic GluR and GABAR subunit mRNAs (Figure 1D). A lower GC content accounts for less stable secondary structures in the 3′-UTRs of GABAR compared to the total, somatic CA1 and neuropil 3′-UTRome as well as to GluR 3′-ends. Interestingly, the cytoplasmic polyadenylation binding element binding protein (CPEB) binds a short, AT-rich sequence within the 3′-UTR of target mRNAs to control translation and to induce the elongation of polyA tails (Mendez and Richter, 2001). By using RNA immunoprecipitation (RIP), it was shown that CPEB1 and 4 bind different GABAR subunits as well as mRNAs coding for scaffold protein such as Gphn (Parras et al., 2018; see also Tables s 1, 2). Moreover, ELAV proteins, among others, bind so-called AU-rich elements (ARE) to stabilize its target mRNAs (Fan and Steitz, 1998; Peng et al., 1998). Therefore, it is tempting to speculate that ELAV proteins also bind mRNAs coding for GABAR subunits to regulate their abundance. Supportive for this idea is an iCLIP-based ELAV target screen from human brain, which detected selective mRNAs encoding GABAR subunits, GABA_B_ receptor auxiliary proteins and GABAR transport proteins (Scheckel et al., 2016; see also Tables s 1, 2).

**Table 1:** Hand-selected list of RBPs with RNAs related to GABAR as targets.

Rbp	Method	Tissue	RNA targets related to GABAR	Reference
Nova	iCLIP	Brain	Gabbr2, Gabrg2	Ule et al. (2003)
FMRP	iCLIP	Brain	Gabbr1, Gabbr2	Darnell et al. (2011)
Staufen1	iCLIP	Brain	Gabbr2	Sugimoto et al. (2015)
Staufen2	RIP, iCLIP	Embryonic brain	Gabra2, Gabra3, Gabbr1, Gabbr2, Gabrb1, Gabrb2, Gabrb3, Gabrg3	Heraud-Farlow et al. (2013) and Sharangdhar et al. (2017)
Unkempt	iCLIP	Embryonic brain	Gabra3, Gabrb2	Murn et al. (2015)
Celf4	iCLIP	Brain	Gabra1, Gabra2, Gabra3, Gabra4, Gabra5, Gabrb1, Gabrb2, Gabrb3, Gabbr1, Gabbr2, Gabrg1, Gabrg2, Gabrg3, Gabrd	Wagnon et al. (2012)
Rbfox1, 2, 3	iCLIP	Brain	Gabra1, Gabra3, Gabra6, Gabbr1, Gabrb2, Gabrb3, Gabrg1, Gabrg2	Lee et al. (2016)
Pumilio1	iCLIP	Brain	Gabra1, Gabra5, Gabbr1, Gabrb2, Gabrg2	Zhang et al. (2017)
Pumilio2	iCLIP	Brain	Gabra4, Gabrb2, Gabrg2, Gabrq	Zhang et al. (2017) and Zahr et al. (2018)
4E-T	RIP	Embryonic brain	Gabrg2	Yang et al. (2014)
hnRNP R	iCLIP	Embryonic primary mouse motorneurons	Gabra4, Gabbr1, Gabrb1, Gabrb3, Gabrg2, Gabrg3	Briese et al. (2018)
CPEB1	RIP	Striatum	Gabrb1, Gabrb2	Parras et al. (2018)
CPEB4	RIP	Striatum	Gabra1, Gabra2, Gabra4, Gabrb1, Gabrb2, Gabrb3, Gabrg3	Parras et al. (2018)
nELAV	iCLIP	Human dorsolateral prefrontal cortex	Gabra4, Gabrb2, Gabrb3, Gabrg1, Gabrg3	Scheckel et al. (2016)

**Table 2:** Hand-selected list of RBPs with RNAs related to scaffold protein, GABAR auxiliary and transport proteins as targets.

Rbp	Method	Tissue	RNA targets related to GABAR	Reference
Nova	iCLIP	Brain	Gphn	Ule et al. (2003)
FMRP	iCLIP	Brain	NSF, Trak2, Ubqln1	Darnell et al. (2011)
Staufen1	iCLIP	Brain	KCTD12, GABARAPL3, NSF, Arfgef2, Ubqln1	Sugimoto et al. (2015)
Staufen2	RIP, iCLIP	Embryonic brain	Gphn, Arhgef9, KCTD16, NSF, Arfgef2, GABARAPL1, Zdhhc3, Plcl1, Ubqln1	Heraud-Farlow et al. (2013) and Sharangdhar et al. (2017)
Rbfox1, 2, 3	iCLIP	Brain	Gphn, NSF, Arfgef2, Ubqln1	Lee et al. (2016)
Pumilio1	iCLIP	Brain	KCTD12, Trak2	Zhang et al. (2017)
Pumilio2	iCLIP	Brain	Gphn, KCTD12, Arfgef2, Trak2, Plcl1	Zhang et al. (2017) and Zahr et al. (2018)
4E-T	RIP	Embryonic brain	Gphn, Trak2	Yang et al. (2014)
hnRNP R	iCLIP	Embryonic primary mouse motorneurons	Gphn, Arhgef9, KCTD16, NSF, Arfgef2, Zdhhc3, Trak2, Plcl1	Briese et al. (2018)
CPEB1	RIP	Striatum	Arfgef2, Zdhhc3	Parras et al. (2018)
CPEB4	RIP	Striatum	Gphn, Arfgef2, Zdhhc3, Trak2, Ubqln1	Parras et al. (2018)
nELAV	iCLIP	Human dorsolateral prefrontal cortex	KCTD16, Plcl1	Scheckel et al. (2016)

To date, several GABAR subunits, scaffold, auxiliary and GABAR transport proteins have been detected as targets for RBPs by iCLIP or RIP (Tables s 1, 2). Among those, known translation regulators such as fragile X mental retardation protein (FMRP), Pumilio1, 2, 4E-T as well as CPEB1 and 4 all bind GABAR subunit mRNAs. However, how these RBPs act together to locally control the expression of GABAR subunits in dendrites is still unknown. Future studies are clearly needed to unravel the role of RBP mediated protein expression control.

## Translation Control: A Possible Regulation of GABA Receptor Protein Abundance and Complex Assembly

Translation is a multistep process that is regulated by versatile proteins (Jackson et al., 2010). Different sequence features of the mRNA that influence translation activity and association with ribosomal polysomes have been characterized in human cell lines (Floor and Doudna, 2016). In detail, the length and structural stability of the 3′-UTR, the number of miRNA binding sites as well as AU elements in the 3′-UTR are main drivers of translation activity located at the 3′-end of the untranslated region. An increase in these features is associated with decreased translation activity in non-neuronal cells (Floor and Doudna, 2016) as well as nerve cells (Blair et al., 2017). For GABAR subunit 3′-UTRs, we observed an increase in 3′-UTR length and AT content (Figures 1A,C,D). These results suggest that translation of these subunits is strongly regulated. Supportive for this idea is the finding that GABAR subunit mRNAs are recognized and subsequently bound by different RBPs (Table 1). In the last decade, several studies revealed that RBPs control translation of their target mRNAs (Hentze et al., 2018). One extensively studied example is the FMRP. FMRP mediated translational control is crucial for neuronal homeostasis and function since loss-of-function leads to severe neurological impairments in synaptic plasticity which cause intellectual disability and social deficits hallmarked for autism spectrum disorders (Bassell and Warren, 2008; Darnell and Klann, 2013). Furthermore, recent studies showed that FMRP is needed for proper differentiation of neuronal stem cells (Castrén et al., 2005; Gao et al., 2018). FMRP has been shown to co-migrate with translationally active ribosomal polysomes (Stefani et al., 2004). However, this finding was challenged by the same study showing that polysomal co-migration is detergent sensitive (Stefani et al., 2004). A mechanistic study combining *in vitro* assays and cryoelectron microscopy reported that FMRP inhibits translation through binding to the ribosomal intersubunit space thereby precluding binding of tRNAs and translation elongation factors (Chen et al., 2014). A transcriptome-wide screen for FMRP targets associated with polysomes identified mRNAs coding for subunits of the GABA_B_ receptor complex (Darnell et al., 2011; see Table 1). Moreover, a recent study showed that the GABA_A_ receptor subunit δ was downregulated in an FMRP knock-out mouse model (Gantois et al., 2006). These findings suggest that FMRP may regulate selected subunits of the GABA_B_ and/or GABA_A_ receptor, most likely at the translational level. Another known translation regulator is Pumilio2 (Pum2). For Pum2, it was shown that it represses translation by competing with the eukaryotic initiation factor (eIF4E) for mRNA 5′-cap binding (Cao et al., 2010), an essential step to start translation initiation (Jackson et al., 2010). Moreover, Pum2 is able to form a complex with the miRNA binding protein Argonaute (Ago) and the eukaryotic translation elongation factor 1A to repress translation elongation (Friend et al., 2014). Next to its role as translation regulator, Pum2 regulates transcript stability through recruitment of the polyA deadenylase complex CCR4-NOT (Van Etten et al., 2012), which is the major protein complex to induce RNA degradation (Collart, 2016). Based on a published iCLIP dataset, Pum2 is able to bind subunits of the GABA_A_ and GABA_B_ receptor (Table 1). Interestingly, double knockdown of Pumilio1 and 2 lead to a decrease in the mRNA levels of certain GABAR subunits (Zhang et al., 2017) indicating that they may be regulated posttranscriptionally by Pumilio proteins. Another RBP that impacts the expression of GABA_A_ receptor subunits, is the non-octamer, POU-domain DNA-binding protein (NONO, also known as p54NRB). NONO belongs to the family of polypyrimidine tract-binding protein-associated splicing factors that are known to regulate various aspects of the RNA lifecycle including transcription regulation, splicing, RNA processing and RNA transport (Yarosh et al., 2015). Interestingly, mutations in the NONO locus causes intellectual disability in humans (Mircsof et al., 2015). Moreover, the authors found that the GABA_A_ receptor-mediated inhibition is mainly affected when NONO is depleted (Mircsof et al., 2015) suggesting that this RBP regulates directly or indirectly the expression of the GABA_A_ receptor. Nonetheless, it is widely unknown which GABAR subunits are translationally regulated. However, the binding of RBPs that are known to control RNA metabolism and translation, clearly suggests the existence of posttranscriptional gene regulation mechanisms for GABARs.

**Figure 2:**
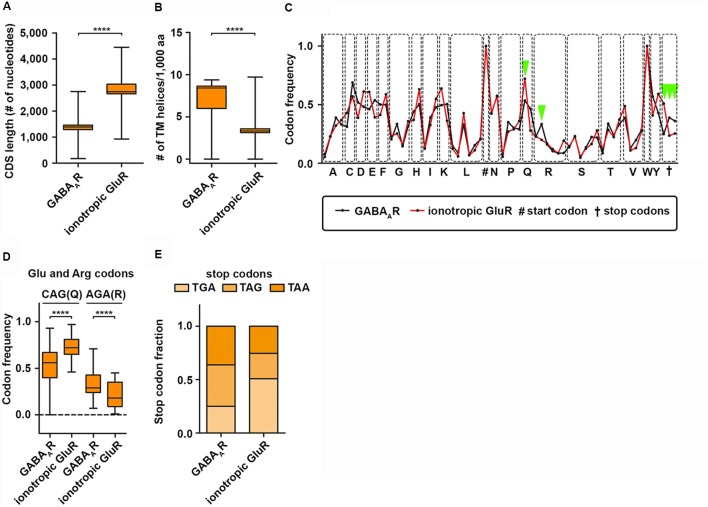
GABA_A_ receptor codon usage differ from ionotropic glutamate receptors. CDS length **(A)** and the number of transmembrane (TM) helices **(B)** in GABA_A_R and ionotropic GluR subunits. **(C)** Codon usage frequency of GABA_A_R and GluR for 20 amino acids and stop codons. Dots represent synonymous codons. **(D)** Codon frequency for CAG (Q) and AGA (R). **(E)** Relative fraction of stop codon usage between GABA_A_R and GluR subunits. Abbreviations: CDS, coding sequence; aa, amino acid. *P*-values were calculated using the Mann-Whitney *U*-test, ****p < 0.0001.

It is commonly accepted that the 3′-UTR allows for translational regulation of mRNAs. Research in the last years, however, has shown that the coding sequence (CDS) can also regulate protein synthesis rate, protein folding and protein complex assembly (Hanson and Coller, 2018). Dynamic translation regulation mediated by the CDS became experimentally accessible with the emergence of deep sequencing technologies and ribosome profiling protocols (Ingolia et al., 2009). Studies in cell lines and cultured neurons revealed that longer CDS are associated with translationally active “heavy” polyribosomes; most likely because a longer CDS can accumulate more ribosomes (Floor and Doudna, 2016; Blair et al., 2017). Interestingly, subunits of the GABA_A_R receptor complex display a shorter CDS compared to ionotropic GluR subunits (Figure 2A) suggestive for differences in translation activity. Another exciting possibility to regulate protein synthesis rate and output is the usage of synonymous codons. Twenty-one amino acids are encoded by 64 codons including three stop codons in the eukaryotic genome (Alberts et al., 2014). This degeneration of the genetic code leads to a codon bias, the preferred usage of certain codons over others to encode the same amino acid. Research in the last decades has shown that the usage bias is not random, but in contrast is driven and influenced by certain features such as translation activity, mRNA stability, protein folding, protein assembly and transcription factor binding (Grantham et al., 1980; Stergachis et al., 2013; Hanson and Coller, 2018). Codons can influence translation speed (Sørensen and Pedersen, 1991) most likely through the levels of cognate and near-cognate tRNAs (Anderson, 1969; Zhang and Ignatova, 2011; Fedyunin et al., 2012; Yu et al., 2015; Hanson and Coller, 2018). Since the nascent chain initiates folding already in the ribosomal exit tunnel (Lu and Deutsch, 2005), the elongation rate can also influence protein folding and, thereby, the protein conformation as it has been shown for the Cystic Fibrosis Transmembrane Regulator (CFTR) in mammalian cells (Kirchner et al., 2017). In line with this finding, Yu et al. (2015) showed using an *in vitro* translation system that codon usage determines co-translational folding through variation in the elongation rate. In particular for a multi-domain protein, it has been suggested that cluster of rare codons flank the parts of the mRNA that code for protein domains. Thus, ribosomes attenuate at these sites allowing the nascent domains to fold first to prevent misfolding (Schieweck et al., 2016; Hanson and Coller, 2018). Protein domains, that are encoded by the downstream mRNA, can then interact with already folded protein substructures to form a functional complex. Moreover, codon usage dependent protein folding can also influence protein specificity, which was reported for the Multi-Drug Resistance 1 protein (MDR1). A silent mutation in a rare codon changes the specificity of MDR1 (Kimchi-sarfaty et al., 2007). Together, these results strongly indicate that dynamics in the translation elongation rate determine trajectories of (co-)translational folding. Based on these results, an intriguing question raises: can codon usage influence protein folding of transmembrane proteins such as subunits of the GABA_A_ receptor? Interestingly, GABA_A_R subunits contain more transmembrane helices compared to ionotropic GluR subunits (Figure 2B). This suggests that GABA_A_R subunits may need more variation in translation speed to allow co-translational folding than ionotropic GluR subunits. Furthermore, GABA_A_R subunits differ in their codon usage compared to GluR subunits (Figure 2C). Overall, the codon usage profiles between the two receptor groups are similar. For some codons, however, we detected significant differences in their frequency (Figures 2D,E). Interestingly, impaired translation of AGA codons leads to neurodegeneration in a mouse model (Ishimura et al., 2014). Moreover, GABA_A_R and GluR subunits exploit different stop codons. While GABA_A_R subunit mRNAs display an almost 1:1:1 ratio, GluR subunits prefer the TGA stop codon that yields the highest readthrough potential in mammalian cell lines (Howard et al., 2000; Bidou et al., 2004; Loughran et al., 2014; Manuvakhova et al., 2014). In addition to co-translational folding, the assembly of large protein complexes can also occur co-translationally (Balchin et al., 2016). It has been shown that this process is crucial for the complex formation in eukaryotic cells (Shiber et al., 2018). It is tempting to speculate that for large neuronal protein complexes such as GABA_A_ receptors, a similar mechanism exists to ensure proper protein-protein interaction. Of note, codon usage and optimality differ dramatically in their impact on RNA stability comparing neurons and non-neuronal cells (Burow et al., 2018). Therefore, a thorough analysis of the neuronal translatome and tRNAome is needed to understand the impact of codon usage on GABA_A_ receptor functioning.

To sum up, findings from different model organisms and cells demonstrate that translation is a highly dynamic process necessary for many aspects of the protein life cycle. For GABA_A_ receptors, it is widely unknown: (i) whether and how they are translationally regulated; and (ii) whether co-translational folding/assembly is necessary for proper GABAR function. However, our bioinformatic predictions suggest that for some aspects, GABAR are prone to be subject to posttranscriptional regulation. Future studies will be clearly needed to unravel the dynamics and regulatory factors of their translation.

## Is Local Protein Synthesis A Prerequsite for Plasticity of Inhibitory Synapses: A Perspective

**Figure 3:**
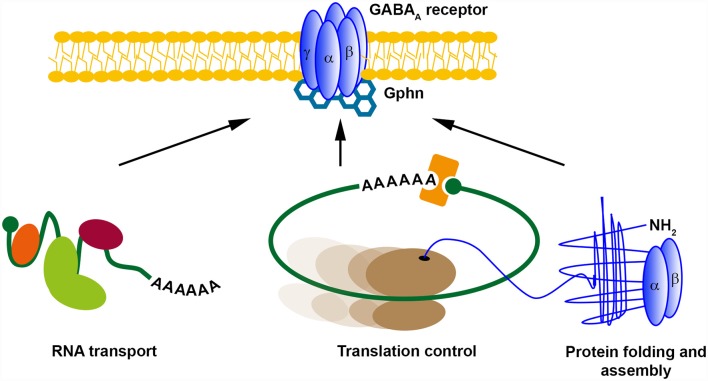
Possible posttranscriptional regulation mechanisms for GABA_A_ receptors. Different posttranscriptional regulatory mechanisms exist. RNA transport, translational control and (co-translational) protein folding and assembly control local protein expression. We propose that GABARs might be regulated at inhibitory synapses in a similar manner. Abbreviation: Gphn, Gephyrin.

Since the discovery of LTP by Bliss and Lomo (1973), numerous studies have unraveled the plasticity of excitatory synapses in the brain aiming to explain the mechanism of learning and memory formation (Kandel et al., 2014). However, how inhibitory synapses undergo structural and molecular plasticity has been widely overlooked for some time (Gaiarsa and Ben-Ari, 2006). One of the first examples that inhibitory synapses show long-term plasticity was a study on Purkinje cells in the cerebellum published in 1998 (Aizenman et al., 1998). Since that time, various studies have addressed the mechanisms of how inhibitory LTP is conveyed (Castillo et al., 2011). Interestingly, in some aspects, inhibitory and excitatory LTP share similar mechanisms including the exchange of synaptic receptors (de Luca et al., 2017) as well as the importance of scaffold proteins for LTP (Petrini et al., 2014). In this context, it was shown that clustering of Gephyrin (Gphn), the major scaffold protein for inhibitory synapses (Tyagarajan and Fritschy, 2014), is essential for GABA_A_ receptor surface dynamics and iLTP (Petrini et al., 2014). In line with its importance for iLTP, Gphn is posttranslationally modified in response to neuronal activity (Flores et al., 2015; Ghosh et al., 2016), which may represent a molecular hub to control inhibitory transmission. Arguably, one of the most impressive examples showing the dynamics of inhibitory synapse formation is the study by Oh et al. (2016). Upon GABA stimulation, newly formed Gphn cluster appear that are the structural basis for inhibitory synapse formation (Tyagarajan and Fritschy, 2014). Based on our bioinformatic predictions (Figures 1, 2) and RBP target screens (Tables s 1, 2), it is tempting to speculate that the appearance of Gphn clusters upon GABA stimulation requires mRNA transport and, subsequently, translation. We propose that these mechanisms are necessary for inhibitory synapse formation (Figure 3). In general, future studies are clearly necessary to address the importance of posttranscriptional gene regulation for GABAergic synaptic transmission. Therefore, it needs to be investigated: (i) which GABAR component is regulated by RBPs; (ii) whether their expression is regulated at the translation, splicing and/or stability level; and (iii) whether their posttranscriptional regulation occurs locally at the synapse. Unraveling the role of RBPs in neuronal inhibition will clearly improve our understanding how neuronal networks are coordinated to find the balance between excitation and inhibition.

## Methods

For analysis, 3′-UTR sequences and length of transmembrane domains were extracted from the EMSEMBL database (genome assembly GRCm38.p6) using the Gene Ontology ID “GO:0016917” for GABARs, “GO:0008066” for glutamate receptors and “GO:0004970” for ionotropic glutamate receptors. Only annotated mRNA isoforms were analyzed. Statistics were calculated using GraphPad Prism (version 5; GraphPad, San Diego, CA, USA).

## Data Availability

All datasets generated for this study are included in the manuscript.

## Author Contributions

RS and MK conceived, executed and discussed the research that is presented in this article. RS generated the figures, the table and wrote the manuscript. RS and MK edited together.

## Conflict of Interest Statement

The authors declare that the research was conducted in the absence of any commercial or financial relationships that could be construed as a potential conflict of interest.
